# Co_2_ and Co_3_ Mixed Cluster Secondary Building Unit Approach toward a Three-Dimensional Metal-Organic Framework with Permanent Porosity

**DOI:** 10.3390/molecules23040755

**Published:** 2018-03-25

**Authors:** Meng-Yao Chao, Wen-Hua Zhang, Jian-Ping Lang

**Affiliations:** College of Chemistry, Chemical Engineering and Materials Science, Soochow University, Suzhou 215123, China; 15062328714@163.com

**Keywords:** metal-organic framework, cobalt cluster, mixed cluster, crystal structure, permanent porosity

## Abstract

Large and permanent porosity is the primary concern when designing metal-organic frameworks (MOFs) for specific applications, such as catalysis and drug delivery. In this article, we report a MOF Co_11_(BTB)_6_(NO_3_)_4_(DEF)_2_(H_2_O)_14_ (**1**, H_3_BTB = 1,3,5-tris(4-carboxyphenyl)benzene; DEF = *N*,*N*-diethylformamide) via a mixed cluster secondary building unit (SBU) approach. MOF **1** is sustained by a rare combination of a linear trinuclear Co_3_ and two types of dinuclear Co_2_ SBUs in a 1:2:2 ratio. These SBUs are bridged by BTB ligands to yield a three-dimensional (3D) non-interpenetrated MOF as a result of the less effective packing due to the geometrically contrasting SBUs. The guest-free framework of **1** has an estimated density of 0.469 g cm^−3^ and exhibits a potential solvent accessible void of 69.6% of the total cell volume. The activated sample of **1** exhibits an estimated Brunauer-Emmett-Teller (BET) surface area of 155 m^2^ g^−1^ and is capable of CO_2_ uptake of 58.61 cm^3^ g^−1^ (2.63 mmol g^−1^, 11.6 wt % at standard temperature and pressure) in a reversible manner at 195 K, showcasing its permanent porosity.

## 1. Introduction

Creating large and permanent porosity in metal-organic frameworks (MOFs) is a prerequisite for particular applications, such as catalysis [1,2,3,4,5,6,7], drug delivery [8,9,10,11,12,13], as well as the study of fundamental host-guest phenomena, including enzyme [14,15,16,17], protein [18], water [19,20,21,22], and nanoparticle [23,24,25,26,27,28,29] encapsulation. However, during the formation of MOFs with targeted porosity, there exists a trade-off between the pore size and framework stability, viz., the shorter ligands generally support stable MOFs with limited porosity, while the longer ligands prefer porous frameworks with limited scaffold stability [30]. For the latter, framework interpenetration often commences as a means to reduce the global energy which manifests Aristotle’s postulation that “Nature abhors a vacuum” [31,32,33,34,35,36].

Chemists are able to solve the interpenetration issue during the synthesis to obtain highly porous MOFs from bulkier ligands, either following the mathematical suggestion [37] or by genuine experimental design [33]. Two factors can be considered from an experimental design perspective, namely, the metal nodes and the ligand struts. From the metal perspective, switching the metal nodes from single metal ion (M), paddle-wheel (M_2_) to bulky clusters, such as pinwheel M_3_ (e.g., MIL-101) [38], tetrahedral M_4_ (e.g., MOF-5) [39], hexanuclear M_6_ (e.g., UiO-66) [40], polymeric [-M-O-] chain SBUs (e.g., MOF-74) [41], among others [42,43], effectively circumvent interpenetration by providing high connectivity of the metal nodes to enhance the steric congestion.

From the ligand perspective, designing an organic ligand alone by increasing its bulkiness to prevent interpenetration and to obtain highly porous MOFs is challenging, and may instead lead to the formation of MOFs with reduced porosity. To this end, two methods are widely practiced to obtain highly porous MOFs. The first method involves the in situ replacement of an existing ligand from the as-synthesized MOF with a similar (usually longer) one, taking advantage of the kinetic lability of the metal-ligand association in solvents [44,45,46,47,48,49,50]. Hupp et al. termed this process as solvent-assisted linker exchange (SALE) [48]. It is notable that even the most robust MOFs, such as UiO-66, MIL-53, and ZIF (e.g., CdIF-4 with RHO topology), are susceptible to SALE in the solution state because of the small energy difference generated by different linker derivatives results in a dynamic situation [49,51,52]. The other more direct approach concerns the use of mixed ligands with different length/angularity but with similar coordination preference to reduce the effective packing of the molecule to achieve non-interpenetrated MOFs with high porosity [53,54].

The limitations of SALE and mix-ligand approach are also obvious. As SALE involves competitive binding of one ligand for the other, usually driven by the difference of the pKa values of the two ligands, the parent MOF should be porous enough to allow the incoming ligands to diffuse into the pores, yet labile enough to allow the competitive binding, and ultimately robust enough to maintain the framework connectivity throughout the exchange process so that the MOFs are not undergoing decomposition or dissolution-recrystallization to yield the undesired complexes [55,56,57]. As for the mix-ligand process, there is a potential risk that each linker forms their own preferred MOFs to achieve the energy minimum of the reaction system [54,58].

By analogy to the mix-ligand approach, the mix-SBU approach also serves to prevent the structure interpenetration to yield stable MOFs with high porosity. An existing and reliable mix-SBU approach is the bottom-up MOF engineering from pre-formed cluster units. For example, Zaworotko et al. [59] adapted the pinwheel [M_3_(*μ*_3_-O)(O_2_CR)_6_] cluster as the SBU to further construct MOFs for targeted applications. Li et al. [60] demonstrated the compatibility of a triangular Cu_3_-pyrazolate SBU with paddle-wheel Zn_2_ and tetrahedral Zn_4_ SBUs to yield a class of mixed-cluster MOFs. These MOFs feature a notable reversible redox switch between Cu^I^_3_(PyC)_3_ and Cu^II^_3_(*μ*-OH)(PyC)_3_(OH)_3_ (H_2_PyC = 4-pyrazolecarboxylic acid) with the retention of the SBU geometry. The Cu_3_ cluster SBU thus functions as an electron reservoir during the catalytic oxidation of CO/aromatic alcohols, and the decomposition of H_2_O_2_.

To this end, the formation of mix SBUs in one MOF remains scarce, presumably because of the usually less compatible geometries of two or more high-connecting SBUs in one MOF. Zhao et al. [61] reported highly porous MOFs respectively sustained by [Cu_12_I_12_] + [Gd_3_] and [Cu_3_I_2_] + [Gd_4_] cluster SBUs for the carboxylation reactions of CO_2_ with terminal alkynes under mild conditions. In this paper, we explore the synthesis of a mix-SBU MOF by using Co(NO_3_)_2_·6H_2_O as the metal source and H_3_BTB as the ligand in DEF (H_3_BTB = 1,3,5-tris(4-carboxyphenyl)benzene; DEF = *N*,*N*-diethylformamide). We chose Co^II^ because the tetrahedral and octahedral Co^2+^ were demonstrated to coexist in equilibrium via the stabilization of oxygen donor ligands [62]. It is therefore not uncommon that the tetrahedral and octahedral Co^2+^ coexist in one SBU of the MOFs [63,64]. We select a large and triangular H_3_BTB as the ligand because it typically forms non-interpenetrated MOFs. DEF is further selected because it is less volatile compared with DMF (*N*,*N*-dimethylformamide) and will guarantee the stability of the MOF for subsequent material characterizations. Thus the one-pot solvothermal reaction of Co(NO_3_)_2_·6H_2_O and H_3_BTB in DEF yielded the 3D MOF of Co_11_(BTB)_6_(NO_3_)_4_(DEF)_2_(H_2_O)_14_ (**1**) in a high yield of 87%. The guest-free framework of **1** has an estimated density of 0.469 g cm^−3^ and features a potentially 69.6% solvent accessible volume. The gas adsorption studies of **1** indicated that it reversibly uptakes CO_2_ over N_2_ and demonstrated permanent porosity.

## 2. Results and Discussion

### 2.1. Synthesis and Material Characterization of MOF ***1***

**Figure 1:**
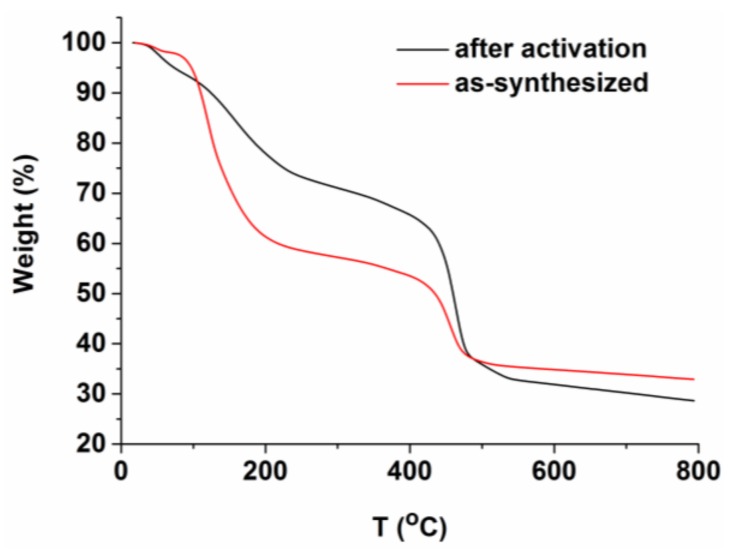
The thermogravimetric analysis (TGA) curves of the as-synthesized and activated samples of metal−organic framework (MOF) **1** showing the continuous weight loss before ca. 500 °C.

MOF **1**, characterized to have a general formula of Co_11_(BTB)_6_(NO_3_)_4_(DEF)_2_(H_2_O)_14_, has been prepared as block purple crystals from a facile one-pot reaction of Co(NO_3_)_2_·6H_2_O and H_3_BTB in DEF under mild reaction conditions (Figure S1). The isolation yield of MOF **1** reaches as high as 87% (based on Co). MOF **1** is stable in MeOH, EtOH, and DMF but experiences a rapid decomposition when immersed in H_2_O. The FT-IR spectrum of **1** contains a sharp peak at 1385 cm^−1^, diagnostic of the presence of NO_3_^−^ [65]. The FT-IR spectrum further features peaks at 2975 cm^−1^, 2930 cm^−1^, and 1643 cm^−1^, corresponding to the presence of –CH_3_, –CH_2_–, and –C=O functionalities and hence the DEF solvate [66]. These observations corroborate the structure deduced from the X-ray crystallographic analysis. The thermogravimetric analysis (TGA) indicates a continuous mass loss of **1** before ca. 500 °C (Figure 1). The first weight loss before 200 °C is likely due to the solvate evaporation, while the second weight loss between 200–500 °C is due to the framework decomposition. Given the large pore percentage (69.6%) of the material and the complexity of solvates included (both coordinated and free DEF and H_2_O), it is not possible to assign the correct ratio of solvents within the pore.

**Figure 2:**
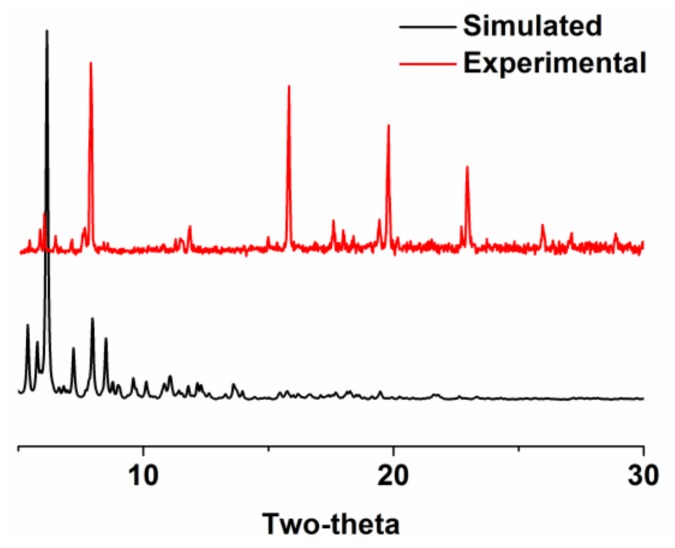
The powder X-ray diffraction (PXRD) of MOF **1** showing the simulated (black) and the experimental (red) patterns.

The powder X-ray diffraction (PXRD) patterns of MOF **1** indicate its crystalline nature with a primary match between the experimental and that of the simulated (Figure 2). However, the intensities of these peaks do not match well. This is typically due to either the different orientation of the crystallites in the bulk material or the pore filling effect [67,68]. The pore filling effect describes that the diffraction intensity deviation (either enhanced or reduced) exists between the solvent-free (simulated) and solvated (experimental) samples [57,68].

### 2.2. Crystal Structure Analysis of MOF ***1***

**Figure 3:**
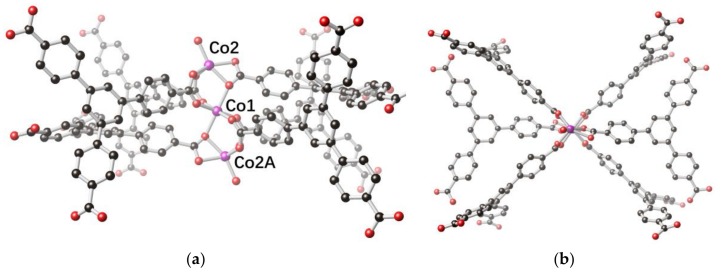

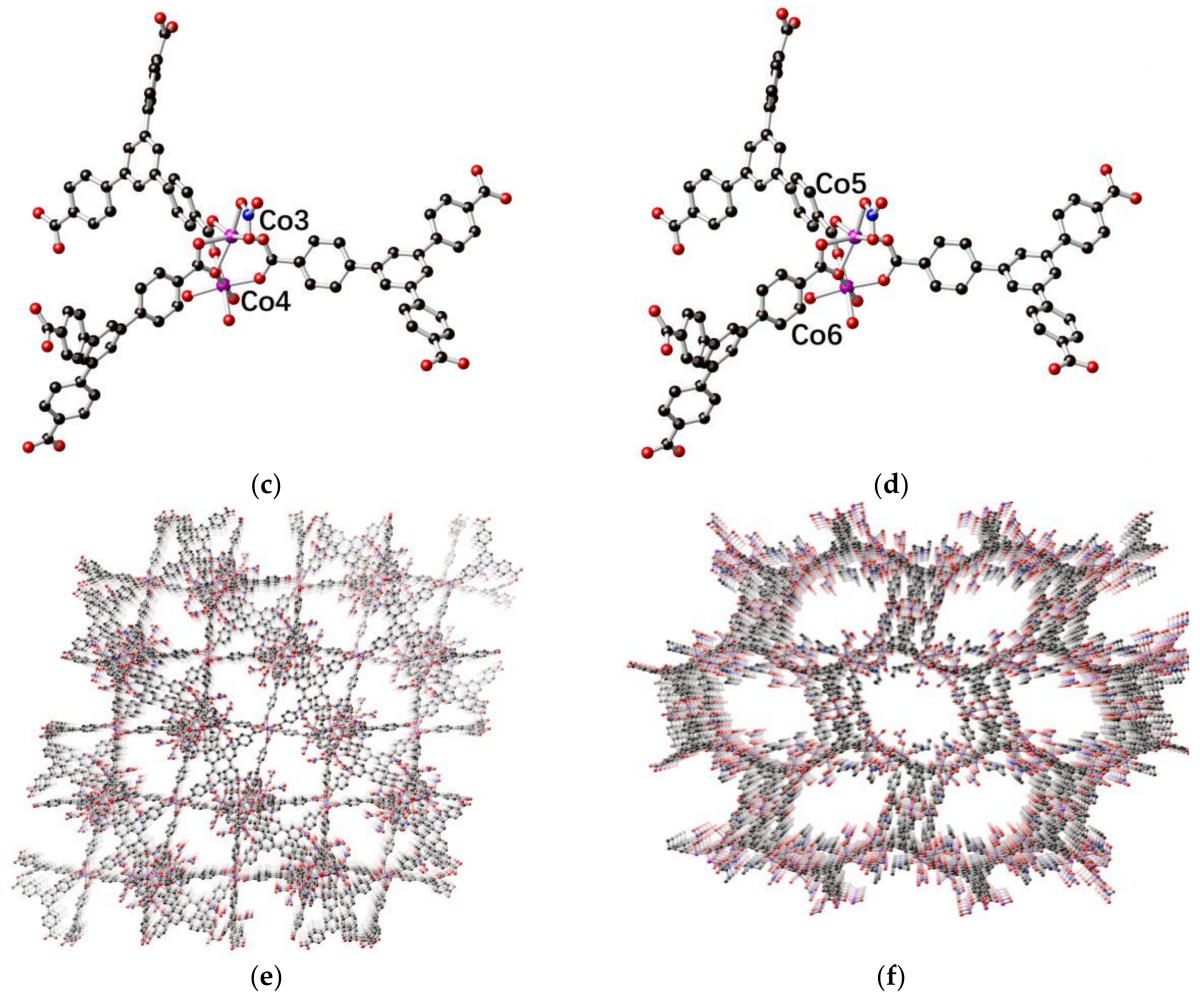
The structure of MOF **1** showing the Co_3_ secondary building unit (SBU) supported by Co1 and Co2 viewing from two directions (**a**,**b**), the Co_2_ SBU supported by Co3 and Co4 (**c**), the Co_2_ SBU supported by Co5 and Co6 (**d**), the 3D network viewing along the [010] direction (**e**), and the 3D network viewing from the crystallographic *a* direction (**f**). All H atoms are omitted. For (**a**–**e**), only one of the disordered portion of the BTB ligand is depicted (H_3_BTB = 1,3,5-tris(4-carboxyphenyl)benzene), and for (**c**,**d**), only one disordered portion of the coordinated anion/solvent is depicted. Color legends: Co (magenta), O (red), N (blue), C (black).

MOF **1** crystallizes in the monoclinic space group *P*2_1_/*n*, and its structure features a Co_3_ cluster and two types of Co_2_ clusters in a 1:2:2 ratio. As shown in Figure 3a, the Co_3_ cluster (based on Co1 and Co2) exhibits a center-of-inversion coincides Co1 and has an hourglass shape. The central Co1 is associated with six O atoms from four bridging COO^−^ (*μ*_2_-*η*^1^:*η*^1^) and two chelating-bridging COO^−^ (*μ*_2_-*η*^1^:*η*^2^) to yield an octahedral coordination with Co1–O bond lengths in the range of 2.048(6)–2.157(6) Å (Table 1). On the other hand, Co2 and its equivalent Co2A (−*x* + 1, −*y* + 1, −*z* + 2) are associated with two O atoms from a pair of bridging COO^−^ (*μ*_2_-*η*^1^:*η*^1^) and a chelating-bridging COO^−^ (*μ*_2_-*η*^1^:*η*^2^), in addition to a terminally coordinated aqua to yield square pyramidal coordination geometries. The Co2–O and Co2A–O bond lengths are in the range of 1.962(6)–2.151(6) Å (Table 1). The bond lengths of Co1–O, Co2–O, and Co2A–O, in turn, suggest that the oxidation states for Co1, Co2, Co2A are +2 instead of +3 which would otherwise give a shorter Co–O bond length of ca. 1.90 Å [69]. The Co–O distances are comparable to those Co_3_ cluster SBUs in MOFs, such as [EMIm]_2_[Co_3_(ip)_4_] (EMIm = 1-ethyl-3-methyl imidazolium; H_2_ip = isophthalic acid) (1.991(4)–2.386(4) Å) [70] and [Co_3_(BPT)_2_(DMF)(bpp)] (H_3_BPT = biphenyl-3,4′,5-tricarboxylic acid, bpp = 1,3-bis(4-pyridyl)propane) (1.978(4)–2.152(3) Å) [71]. Each Co_3_ SBU is thus coordinated to six BTB ligands, which in turn further associated with 12 Co_2_ SBUs, of which six are based on Co3 and Co4, and the other six based on Co5 and Co6. It is notable that among the six BTB ligands associated with the Co_3_ SBU, two are functioning as the in-plane ligands propagating the structure within the (101) plane, while the other four are largely responsible for the epitaxial propagation of the structure along the [010] direction (Figure 3a,b,e).

The dinuclear Co_2_ SBUs based on Co3 and Co4, Co5 and Co6 are similar except the terminal coordinated solvates (Figure 3c,d). In each of the Co_2_ SBU, a pair of Co atoms are bridged by two bridging COO^−^ (*μ*_2_-*η*^1^:*η*^1^) and one chelating-bridging COO^-^ (*μ*_2_-*η*^1^:*η*^2^). Apart from this similarity, Co3–Co6 are further bonded to disordered NO_3_^−^/H_2_O (Co3 and Co4), NO_3_^−^/H_2_O/DEF (Co5 and Co6) (see X-ray crystallography section for detailed disorder manipulation). Each Co_2_ SBU thus extended to six cluster SBUs, including three Co_3_, two equivalent Co_2_ SBUs, as well as one alternative Co_2_ SBU. From a topological sense, the Co_3_ SBU serves as a 6-connecting node, while the two types of Co_2_ and three independent BTB ligands all serve as 3-connecting nodes. The Co_3_ and two types of Co_2_ SBUs are interconnected by BTB ligands to give a complexed 3D non-interpenetrated structure with the topological symbol of (6^2^∙8^6^∙10^6^∙12)_Co3_(6∙8^2^)_Co2_(6∙8^2^)_Co2_(6∙8^2^)_BTB_(6∙8∙10)_BTB_(6∙8∙10)_BTB_ as suggested by the OLEX program (Figure S3) [72]. It should be noted that compositionally similar Co-BTB MOFs featuring mixed Co_3_ and Co_2_ cluster SBUs are reported but with totally different topology [73]. When looking along the [010] direction (Figure 3e) and the crystallographic *a* direction (Figure 3f), small parallelogram and large oval-shaped pores are feasible. These pore volumes sum up to 19,398.6 Å^3^ per unit cell, which occupied 69.6% of the total cell volume (27,861.0 Å^3^) as calculated by the Platon program [74]. As a result, a guest-free scaffold of MOF **1** features a low density of 0.469 g cm^−3^ (Table 2).

### 2.3. Gas Adsorption Behavior of MOF ***1***

**Figure 4:**
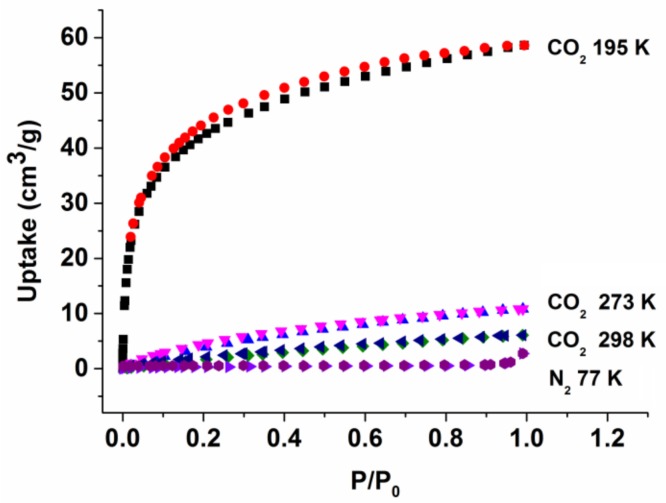
N_2_ (77 K) and CO_2_ (195 K, 273 K, and 298 K) sorption isotherms for MOF **1** with pressures of up to 1 bar. The black square and red circle represent CO_2_ absorption and desorption at 195 K, the blue and pink triangles represent CO_2_ absorption and desorption at 273 K, the green square and the dark blue triangle represent CO_2_ absorption and desorption at 298 K, while the purple triangle and dark purple hexagon represent N_2_ absorption and desorption. P_0_ is the saturated vapor pressure of the adsorbates at the measurement temperatures.

The pore surfaces of MOF **1** are decorated with charged/polar NO_3_^−^/H_2_O/DEF, an ideal trait of an absorbent for N_2_ and CO_2_ with quadruple moments of 1.52 × 10^−26^ esu cm^2^ and 4.30 × 10^−26^ esu cm^2^ [75,76]. The sample activation was guided by the TGA results (Figure 1). To maintain the framework integrity of MOF **1** for subsequent porosity measurement, the sample activation temperature and pressure were set to 323 K and 10^−^^2^ kPa. The solvents in the pores were quickly exchanged with CHCl_3_ twice before transferring to the instrument for further activation. We observed no obvious absorption of N_2_ at 77 K (Figure 4) but notable uptake of CO_2_ at 195 K for MOF **1**. This presumably results from the larger kinetic size of N_2_ (3.64 Å) than CO_2_ (3.30 Å) [75,77]. In addition, the surface crack of the crystals is also a potential limiting factor for the differentiation of gas uptake, as demonstrated by Matzger et al. [78,79] using the positron annihilation lifetime spectroscopy. Furthermore, CO_2_ exhibits a significant quadrupole moment than N_2_, which makes it more susceptible to induce interactions with MOFs, such as association with open metal sites, involving in hydrogen bonding, etc. [80]. In our case, we found that the TGA of the activated sample exhibits similar scaffold decomposition temperature as that of the as-synthesized (Figure 1), but its PXRD pattern becomes amorphous (Figure S2), suggesting some collapse of the crystal under the activation condition and may ultimately lead to the reduction of the pore size to exclude the N_2_ uptake. MOF **1** exhibited a type I CO_2_ adsorption profile with steep uptake in the low-pressure region, typical of a microporous material with open channels in the degassed phase [45,81,82,83]. The CO_2_ adsorption isotherm for MOF **1** exhibits a negligible hysteresis upon desorption, an indication of the scaffold rigidity. While being fully aware that N_2_ is the most utilized probe molecule to estimate the BET surface areas, other probes, such as Ar and CO_2_, may also serve as useful alternative to determine the BET surface areas, particularly for those MOFs featuring ultramicropores [84,85]. The Brunauer-Emmett-Teller (BET) surface area of MOF **1** can be roughly estimated based on the CO_2_ adsorption data in the linear range (P/P_0_ < 0.07) to be 155 m^2^ g^−1^. MOF **1** also adsorbs up to 58.61 cm^3^ g^−1^ CO_2_ (2.63 mmol g^−1^, 11.6 wt % at standard temperature and pressure, STP), comparable with the respective data found for the 3D MOFs of [Zn(*iso*-hmn)] (1.63 mmol g^−1^; 7.23 wt % at STP; *iso*-hmn = 2-(hydroxymethyl)isonicotinate) [81], [Cd_3_(BTB)_2_(bpe)(H_2_O)_2_] (3.94 mmol g^−1^; 17.3 wt % at STP; bpe = *trans*-1,2-bis(4-pyridyl)ethylene) [45], and Mn(2,6-ndc) (3.0 mmol g^−1^; 13.0 wt % at STP; ndc = 2,6-naphthalenedicarboxylate) [86]. It is also notable that the activated samples of MOF **1** exhibit nearly no CO_2_ uptake at 273 K and 298 K (Figure 4).

## 3. Synthesis and Material Characterization

### 3.1. General

Co(NO_3_)_2_·6H_2_O (Sinopharm Chemical Reagent Co., Ltd., Shanghai, China, ≥98.5%), benzene-1,3,5-tribenzoic acid (H_3_BTB, >98.0%, TCI), *N*,*N*-diethylformamide (DEF, >99.0%, TCI) were commercially available and used without further purification. IR spectrum was measured on a Varian 1000 FT-IR spectrometer (Varian, Inc., Palo Alto, CA, USA) as KBr disks (400–4000 cm^−1^). Elemental analyses for C, H, and N were carried out on a Carlo-Erba CHNO-S microanalyzer (Carlo Erba, Waltham, MA, USA). The Thermogravimetric Analyzer was used at a heating rate of 5 °C min^−1^ under a nitrogen gas flow in an Al_2_O_3_ pan. Powder X-ray diffraction (PXRD) patterns were recorded with a Bruker D8 GADDS (General Area Detector Diffraction System) micro-diffractometer (Bruker AXS GmbH, Karlsruhe, Germany) equipped with a VANTEC-2000 area detector (Bruker AXS GmbH, Germany) with Φ rotation method. The gas adsorption-desaorption isotherms were recorded using BELSORP-max (MicrotracBEL Corp., Osaka, Japan). The sample was air dried in the fume hood and exchanged with CHCl_3_ twice before transferring to the instrument for activation. The sample was heated to proper temperature under a vacuum of 10^−2^ kPa for 36 h. The evacuated sample tube was weighed again, and the sample mass was determined by subtracting the mass of the previously. The N_2_ and CO_2_ isotherms were measured using a liquid nitrogen bath (77 K), dry ice/acetone (195 K), and cooling machines EtOH/H_2_O (*v*/*v* = 1:1) as coolant (273 K and 298 K), respectively.

### 3.2. Synthesis of Co_11_(BTB)_6_(NO_3_)_4_(DEF)_2_(H_2_O)_14_ (MOF ***1***)

A mixture of Co(NO_3_)_2_·6H_2_O (34.8 mg, 0.12 mmol) and H_3_BTB (35.2 mg, 0.067 mmol) in 6 mL DEF was placed into a 15 mL glass tube and sealed. The tube was transferred into a programmed oven and heated from 25 °C to 85 °C over 4 h and then maintained at this temperature for 72 h before slow cooling to 25 °C (48 h from 85 °C to 25 °C) to yield purple crystals (37.5 mg, 87% based on Co). IR (400–4000 cm^−1^, KBr disks): 3427 (s), 2975 (vw), 1643 (s), 1608 (s), 1541 (m), 1400 (vs), 1385 (vs), 1125 (m), 1017 (w), 855 (w), 780 (m), 706 (w), 617 (w), 505 (vw). Anal. Calcd. (%) for Co_11_(BTB)_6_(NO_3_)_4_(DEF)_2_(H_2_O)_14_ (after activation): C 52.12, H 3.54, N 2.12; found: C 56.28, H 5.62, N 3.41.

### 3.3. X-Ray Crystallography for MOF ***1***

The single crystal of MOF **1** was analyzed on a Bruker D8 Quest CCD X-ray diffractometer with graphite monochromated Mo Kα (λ = 0.71073 Å) radiation. Refinement and reduction of the collected data were achieved by the program Bruker SAINT with absorption correction (multi-scan) applied [87]. The structure was solved by direct method and refined on *F*^2^ by full-matrix least-squares techniques with SHELXTL-2013 [88]. In **1**, each BTB ligand contains one disordered phenyl group with relative ratios of 0.56/0.44, 0.56/0.44, and 0.60/0.40 refined for the two components. The Co3 coordination site is associated with 0.25 NO_3_^−^ (contains N3)/0.75 H_2_O, and 0.33 NO_3_^−^ (contains N4)/0.67 H_2_O. The Co4 coordination site is associated with 0.5 NO_3_^−^ (contains N1)/0.25 NO_3_^−^ (contains N2)/0.25 H_2_O. Similar to that for Co3, each of the coordination site for Co5 and Co6 is associated with 0.33 NO_3_^−^ (contains N4)/0.67 H_2_O. In addition, each coordination site for both Co5 and Co6 is also associated with 0.50 DEF /0.50 H_2_O. A large amount of spatially delocalized electron density (1408 electrons) in the lattice was found but acceptable refinement results could not be obtained for this electron density. The solvent contribution was then modeled using SQUEEZE in the Platon program suite [89]. Crystallographic data for MOF **1** has been deposited in the Cambridge Crystallographic Data Center (CCDC) as supplementary publication number of 1,826,274. These data can be obtained free of charge either from the CCDC via www.ccdc.cam.ac.uk/data_request/cif (or from the CCDC, 12 Union Road, Cambridge CB2 1EZ, UK; Fax: +44 1223 336033; E-mail: deposit@ccdc.cam.ac.uk) or from the Supporting Information. A summary of the key crystallographic data is listed in Table 1, and the selected bond lengths are listed in Table 2.

## 4. Conclusions

In summary, we have prepared and characterized a porous 3D MOF (MOF **1**) sustained by a Co_3_ and two types of Co_2_ mixed cluster SBUs. The activated sample of MOF **1** exhibits CO_2_ gas uptake at 195 K and permanent porosity. In contrast, MOF **1** showed negligible N_2_ uptake at 77 K, as well as CO_2_ at 273 K and 295 K, indicating the collapse of the MOF scaffold, probably due to the improper activation. In our future work, we will employ a more effective activation method such as supercritical CO_2_, to obtain MOFs with high surface areas. The ionic nature of MOF **1** with solvent-like or weakly associated NO_3_^−^ in its pores coupled with its wide pore opening may further allow solution-based anion exchange for the removal of environmentally relevant anions, such as TcO_4_^−^ [90], Cr_2_O_7_^2−^ [91], induced by better size match between these anions and the host framework. We are currently exploring this anion exchange possibility of MOF **1**.

## Figures and Tables

**Table 1:** Crystallographic data and refinement parameters for MOF **1**.

Parameter	Value
Molecular formula	C_172_H_112_Co_11_N_6_O_64_
Formula weight	3934.90
Crystal system	Monoclinic
Space group	*P*2_1_/*n*
*a* (Å)	27.295(3)
*b* (Å)	36.845(4)
*c* (Å)	29.289(3)
*β* (°)	108.940(2)
*V* (Å^3^)	27,861(5)
Z	2
*ρ*_calc_ (g cm^−3^)	0.469
*F*(000)	3990
*µ* (mm^−1^)	0.347
Total reflections	483,464
Unique reflections	49,039
Observed reflections	21,553
*R*_int_	0.1868
Variables	1138
*R*_1_ *^a^*	0.1225
*wR*_2_ *^b^*	0.2201
GOF *^c^*	1.130
*ρ*_max_/*ρ*_min_(e Å^−3^)	1.864/−2.042

*^a^ R*_1_ = Σ||F_o_| − |F_c_||/Σ|F_o_|, *^b^ wR*_2_ = {Σ[*w*(F_o_^2^ − F_c_^2^)^2^]/Σ[*w*(F_o_^2^)^2^]}^1/2^, *^c^* GOF = {Σ[*w*(F_o_^2^ − F_c_^2^)^2^]/(*n* − *p*)}^1/2^, where *n* is the number of reflections, and *p* is total number of parameters refined.

**Table 2:** Selected bond distances of MOF **1** involving the Co centers.

Co(1)–O(1)#1	2.048(6)	Co(1)–O(1)	2.048(6)	Co(1)–O(7)	2.077(5)
Co(1)–O(7)#1	2.077(5)	Co(1)–O(13)	2.157(6)	Co(1)–O(13)#1	2.157(6)
Co(2)–O(2)#1	1.962(6)	Co(2)–O(8)	1.984(5)	Co(2)–O(22)	2.051(5)
Co(2)–O(14)	2.122(5)	Co(2)–O(13)	2.151(6)	Co(3)–O(12)#2	2.034(5)
Co(3)–O(17)#3	2.056(5)	Co(3)–O(30)	2.082(7)	Co(3)–O(9)	2.093(5)
Co(3)–O(26)	2.104(6)	Co(3)–O(34)	2.132(7)	Co(4)–O(11)#2	1.989(6)
Co(4)–O(18)#3	1.994(5)	Co(4)–O(19)	2.102(7)	Co(4)–O(10)	2.136(4)
Co(4)–O(9)	2.186(4)	Co(4)–O(46)	2.20(3)	Co(4)–O(20)	2.228(7)
Co(5)–O(5)#4	2.007(5)	Co(5)–O(15)	2.060(5)	Co(5)–O(3)#5	2.077(5)
Co(5)–O(44)	2.082(6)	Co(5)–O(35)	2.100(7)	Co(5)–O(43)	2.195(8)
Co(6)–O(16)	2.032(5)	Co(6)–O(6)#4	2.048(5)	Co(6)–O(45)	2.059(6)
Co(6)–O(3)#5	2.137(5)	Co(6)–O(39)	2.149(7)	Co(6)–O(4)#5	2.236(4)

Symmetry transformations used to generate equivalent atoms: #1 –*x* + 1, −*y* + 1, −*z* + 2; #2 *x* − 1/2, −*y* + 3/2, *z* − 1/2; #3 *x* − 1, *y*, *z*; #4 –*x* + 2, −*y* + 1, −*z* + 2; #5 –*x* + 3/2, *y* − 1/2, −*z* + 3/2; #6 −*x* + 3/2, *y* + 1/2, −*z* + 3/2; #7 *x* + 1/2, −*y* + 3/2, *z* + 1/2; #8 *x* + 1, *y*, *z*.

## Supplementary Materials

The following are available online. Figure S1: The photo of as-synthesized MOF **1** crystals. Figure S2: The PXRD pattern of MOF **1** after BET test showing its amorphous nature., Figure S3: The topological network of MOF **1** by considering the Co_3_ SBU as a 6-connecting node, while the two types of Co_2_ SBUs, as well as three independent BTB ligands as 3-connecting nodes.

## References

[1] Ikuno T., Zheng J., Vjunov A., Sanchez-Sanchez M., Ortuño M.A., Pahls D.R., Fulton J.L., Camaioni D.M., Li Z., Ray D. (2017). Methane oxidation to methanol catalyzed by Cu-oxo clusters stabilized in NU-1000 metal-organic framework. J. Am. Chem. Soc..

[2] Chen Y.-Z., Wang Z.U., Wang H., Lu J., Yu S.-H., Jiang H.-L. (2017). Singlet oxygen-engaged selective photo-oxidation over Pt nanocrystals/porphyrinic MOF: The roles of photothermal effect and Pt electronic state. J. Am. Chem. Soc..

[3] Burgun A., Coghlan C.J., Huang D.M., Chen W., Horike S., Kitagawa S., Alvino J.F., Metha G.F., Sumby C.J., Doonan C.J. (2017). Mapping-out catalytic processes in a metal-organic framework with single-crystal X-ray crystallography. Angew. Chem. Int. Ed..

[4] Zhang T., Manna K., Lin W.B. (2016). Metal-organic frameworks stabilize solution-inaccessible cobalt catalysts for highly efficient broad-scope organic transformations. J. Am. Chem. Soc..

[5] Zhang H., Wei J., Dong J., Liu G., Shi L., An P., Zhao G., Kong J., Wang X., Meng X. (2016). Efficient visible-light-driven carbon dioxide reduction by a single-atom implanted metal-organic framework. Angew. Chem. Int. Ed..

[6] Liu L., Harris T.D. (2016). Metal-organic frameworks as potential catalysts for industrial 1-butene production. ACS Cent. Sci..

[7] Ikemoto K., Inokuma Y., Rissanen K., Fujita M. (2014). X-ray snapshot observation of palladium-mediated aromatic bromination in a porous complex. J. Am. Chem. Soc..

[8] Zhuang J., Young A.P., Tsung C.-K. (2017). Integration of biomolecules with metal-organic frameworks. Small.

[9] Wu M.-X., Yang Y.-W. (2017). Metal-organic framework (MOF)-based drug/cargo delivery and cancer therapy. Adv. Mater..

[10] Teplensky M.H., Fantham M., Li P., Wang T.C., Mehta J.P., Young L.J., Moghadam P.Z., Hupp J.T., Farha O.K., Kaminski C.F. (2017). Temperature treatment of highly porous zirconium-containing metal-organic frameworks extends drug delivery release. J. Am. Chem. Soc..

[11] Chen Q., Xu M., Zheng W., Xu T., Deng H., Liu J. (2017). Se/Ru-decorated porous metal-organic framework nanoparticles for the delivery of pooled siRNAs to reversing multidrug resistance in taxol-resistant breast cancer cells. ACS Appl. Mater. Interfaces.

[12] Zheng H., Zhang Y., Liu L., Wan W., Guo P., Nyström A.M., Zou X. (2016). One-pot synthesis of metal-organic frameworks with encapsulated target molecules and their applications for controlled drug delivery. J. Am. Chem. Soc..

[13] Inokuma Y., Yoshioka S., Ariyoshi J., Arai T., Hitora Y., Takada K., Matsunaga S., Rissanen K., Fujita M. (2013). X-ray analysis on the nanogram to microgram scale using porous complexes. Nature.

[14] Lian X., Fang Y., Joseph E., Wang Q., Li J., Banerjee S., Lollar C., Wang X., Zhou H.-C. (2017). Enzyme-MOF (metal-organic framework) composites. Chem. Soc. Rev..

[15] Lian X., Erazo-Oliveras A., Pellois J.-P., Zhou H.-C. (2017). High efficiency and long-term intracellular activity of an enzymatic nanofactory based on metal-organic frameworks. Nat. Commun..

[16] Zhao X., Mao C., Luong K.T., Lin Q., Zhai Q.-G., Feng P., Bu X. (2016). Framework cationization by preemptive coordination of open metal sites for anion-exchange encapsulation of nucleotides and coenzymes. Angew. Chem. Int. Ed..

[17] Li P., Modica J.A., Howarth A.J., Vargas L E., Moghadam P.Z., Snurr R.Q., Mrksich M., Hupp J.T., Farha O.K. (2016). Toward design rules for enzyme immobilization in hierarchical mesoporous metal-organic frameworks. Chem.

[18] Williams D.E., Dolgopolova E.A., Pellechia P.J., Palukoshka A., Wilson T.J., Tan R., Maier J.M., Greytak A.B., Smith M.D., Krause J.A. (2015). Mimic of the green fluorescent protein β-barrel: Photophysics and dynamics of confined chromophores defined by a rigid porous scaffold. J. Am. Chem. Soc..

[19] Towsif Abtab S.M., Alezi D., Bhatt P.M., Shkurenko A., Belmabkhout Y., Aggarwal H., Weseliński Ł.J., Alsadun N., Samin U., Hedhili M.N. (2018). Reticular chemistry in action: A hydrolytically stable MOF capturing twice its weight in adsorbed water. Chem.

[20] Kim H., Yang S., Rao S.R., Narayanan S., Kapustin E.A., Furukawa H., Umans A.S., Yaghi O.M., Wang E.N. (2017). Water harvesting from air with metal-organic frameworks powered by natural sunlight. Science.

[21] Cadiau A., Belmabkhout Y., Adil K., Bhatt P.M., Pillai R.S., Shkurenko A., Martineau-Corcos C., Maurin G., Eddaoudi M. (2017). Hydrolytically stable fluorinated metal-organic frameworks for energy-efficient dehydration. Science.

[22] Deng H., Grunder S., Cordova K.E., Valente C., Furukawa H., Hmadeh M., Gandara F., Whalley A.C., Liu Z., Asahina S. (2012). Large-pore apertures in a series of metal-organic frameworks. Science.

[23] Kim I.S., Li Z., Zheng J., Platero-Prats A.E., Mavrandonakis A., Pellizzeri S., Ferrandon M., Vjunov A., Gallington L.C., Webber T.E. (2018). Sinter-resistant platinum catalyst supported by metal-organic framework. Angew. Chem. Int. Ed..

[24] Zanon A., Verpoort F. (2017). Metals@ZIFs: Catalytic applications and size selective catalysis. Coord. Chem. Rev..

[25] Yang Q., Xu Q., Jiang H.-L. (2017). Metal-organic frameworks meet metal nanoparticles: Synergistic effect for enhanced catalysis. Chem. Soc. Rev..

[26] Lismont M., Dreesen L., Wuttke S. (2017). Metal-organic framework nanoparticles in photodynamic therapy: Current status and perspectives. Adv. Funct. Mater..

[27] Fortea-Pérez F.R., Mon M., Ferrando-Soria J., Boronat M., Leyva-Pérez A., Corma A., Herrera J.M., Osadchii D., Gascon J., Armentano D. (2017). The MOF-driven synthesis of supported palladium clusters with catalytic activity for carbene-mediated chemistry. Nat. Mater..

[28] Zhu Q.-L., Xu Q. (2016). Immobilization of ultrafine metal nanoparticles to high-surface-area materials and their catalytic applications. Chem.

[29] Yang Q., Xu Q., Yu S.-H., Jiang H.-L. (2016). Pd nanocubes@ZIF-8: Integration of plasmon-driven photothermal conversion with a metal-organic framework for efficient and selective catalysis. Angew. Chem. Int. Ed..

[30] Wang X.-S., Ma S., Sun D., Parkin S., Zhou H.-C. (2006). A mesoporous metal-organic framework with permanent porosity. J. Am. Chem. Soc..

[31] Ferguson A., Liu L., Tapperwijn S.J., Perl D., Coudert F.-X., Van Cleuvenbergen S., Verbiest T., van der Veen M.A., Telfer S.G. (2016). Controlled partial interpenetration in metal-organic frameworks. Nat. Chem..

[32] Jiang H.-L., Makala T.A., Zhou H.-C. (2013). Interpenetration control in metal-organic frameworks for functional applications. Coord. Chem. Rev..

[33] Zhang J., Wojtas L., Larsen R.W., Eddaoudi M., Zaworotko M.J. (2009). Temperature and concentration control over interpenetration in a metal-organic material. J. Am. Chem. Soc..

[34] Liu Q., Ren Z.G., Deng L., Zhang W.H., Zhao X., Sun Z.R., Lang J.P. (2015). Solvent effect-driven assembly of W/Cu/S cluster-based coordination polymers from the cluster precursor [Et_4_N][Tp*WS_3_(CuBr)_3_] and CuCN: Isolation, structures and enhanced NLO responses. Dalton Trans..

[35] Zhang W.H., Ren Z.G., Lang J.P. (2016). Rational construction of functional molybdenum (tungsten)-copper-sulfur coordination oligomers and polymers from preformed cluster precursors. Chem. Soc. Rev..

[36] Aggarwal H., Bhatt P.M., Bezuidenhout C.X., Barbour L.J. (2014). Direct evidence for single-crystal to single-crystal switching of degree of interpenetration in a metal-organic framework. J. Am. Chem. Soc..

[37] Delgado-Friedrichs O., O’Keeffe M. (2007). Three-periodic tilings and nets: Face-transitive tilings and edge-transitive nets revisited. Acta Crystallogr. Sect. A.

[38] Férey G., Mellot-Draznieks C., Serre C., Millange F., Dutour J., Surble S., Margiolaki I. (2005). A chromium terephthalate-based solid with unusually large pore volumes and surface area. Science.

[39] Li H., Eddaoudi M., O’Keeffe M., Yaghi O.M. (1999). Design and synthesis of an exceptionally stable and highly porous metal-organic framework. Nature.

[40] Cavka J.H., Jakobsen S., Olsbye U., Guillou N., Lamberti C., Bordiga S., Lillerud K.P. (2008). A new zirconium inorganic building brick forming metal organic frameworks with exceptional stability. J. Am. Chem. Soc..

[41] Caskey S.R., Wong-Foy A.G., Matzger A.J. (2008). Dramatic tuning of carbon dioxide uptake via metal substitution in a coordination polymer with cylindrical pores. J. Am. Chem. Soc..

[42] Farha O.K., Eryazici I., Jeong N.C., Hauser B.G., Wilmer C.E., Sarjeant A.A., Snurr R.Q., Nguyen S.T., Yazaydın A.Ö., Hupp J.T. (2012). Metal-organic framework materials with ultrahigh surface areas: Is the sky the limit?. J. Am. Chem. Soc..

[43] Li J.-R., Timmons D.J., Zhou H.-C. (2009). Interconversion between molecular polyhedra and metal-organic frameworks. J. Am. Chem. Soc..

[44] Dutta A., Wong-Foy A.G., Matzger A.J. (2014). Coordination copolymerization of three carboxylate linkers into a pillared layer framework. Chem. Sci..

[45] Zhang Z.X., Ding N.N., Zhang W.H., Chen J.X., Young D.J., Hor T.S.A. (2014). Stitching 2D polymeric layers into flexible interpenetrated metal-organic frameworks within single crystals. Angew. Chem. Int. Ed..

[46] Liu C., Zeng C., Luo T.-Y., Merg A.D., Jin R., Rosi N.L. (2016). Establishing porosity gradients within metal-organic frameworks using partial postsynthetic ligand exchange. J. Am. Chem. Soc..

[47] Deria P., Mondloch J.E., Karagiaridi O., Bury W., Hupp J.T., Farha O.K. (2014). Beyond post-synthesis modification: Evolution of Metal-organic frameworks via building block replacement. Chem. Soc. Rev..

[48] Karagiaridi O., Bury W., Mondloch J.E., Hupp J.T., Farha O.K. (2014). Solvent-assisted linker exchange: An alternative to the de novo synthesis of unattainable metal-organic frameworks. Angew. Chem. Int. Ed..

[49] Kim M., Cahill J.F., Fei H., Prather K.A., Cohen S.M. (2012). Postsynthetic ligand and cation exchange in robust metal-organic frameworks. J. Am. Chem. Soc..

[50] Burnett B.J., Barron P.M., Hu C., Choe W. (2011). Stepwise synthesis of metal-organic frameworks: Replacement of structural organic linkers. J. Am. Chem. Soc..

[51] Al-Maythalony B.A., Alloush A.M., Faizan M., Dafallah H., Elgzoly M.A.A., Seliman A.A.A., Al-Ahmed A., Yamani Z.H., Habib M.A.M., Cordova K.E. (2017). Tuning the interplay between selectivity and permeability of ZIF-7 mixed matrix membranes. ACS Appl. Mater. Interfaces.

[52] Karagiaridi O., Bury W., Sarjeant A.A., Stern C.L., Farha O.K., Hupp J.T. (2012). Synthesis and characterization of isostructural cadmium zeolitic imidazolate frameworks via solvent-assisted linker exchange. Chem. Sci..

[53] Liu L., Zhou T.-Y., Telfer S.G. (2017). Modulating the performance of an asymmetric organocatalyst by tuning its spatial environment in a metal-organic framework. J. Am. Chem. Soc..

[54] Liu L., Telfer S.G. (2015). Systematic ligand modulation enhances the moisture stability and gas sorption characteristics of quaternary metal-organic frameworks. J. Am. Chem. Soc..

[55] Thorp-Greenwood F.L., Kulak A.N., Hardie M.J. (2015). An infinite chainmail of M_6_L_6_ metallacycles featuring multiple borromean links. Nat. Chem..

[56] Hirai K., Reboul J., Morone N., Heuser J.E., Furukawa S., Kitagawa S. (2014). Diffusion-coupled molecular assembly: Structuring of coordination polymers across multiple length scales. J. Am. Chem. Soc..

[57] Kimitsuka Y., Hosono E., Ueno S., Zhou H., Fujihara S. (2013). Fabrication of porous cubic architecture of ZnO using Zn-terephthalate MOFs with characteristic microstructures. Inorg. Chem..

[58] Koh K., Wong-Foy A.G., Matzger A.J. (2008). A crystalline mesoporous coordination copolymer with high microporosity. Angew. Chem. Int. Ed..

[59] Schoedel A., Boyette W., Wojtas L., Eddaoudi M., Zaworotko M.J. (2013). A family of porous lonsdaleite-e networks obtained through pillaring of decorated kagomé lattice sheets. J. Am. Chem. Soc..

[60] Tu B., Pang Q., Xu H., Li X., Wang Y., Ma Z., Weng L., Li Q. (2017). Reversible redox activity in multicomponent metal-organic frameworks constructed from trinuclear copper pyrazolate building blocks. J. Am. Chem. Soc..

[61] Xiong G., Yu B., Dong J., Shi Y., Zhao B., He L.-N. (2017). Cluster-based MOFs with accelerated chemical conversion of CO_2_ through C-C bond formation. Chem. Commun..

[62] Aizawa S.-I., Funahashi S. (2002). Octahedral-tetrahedral equilibrium and solvent exchange of cobalt(II) ions in primary alkylamines. Inorg. Chem..

[63] Yu C., Ma S., Pechan M.J., Zhou H.-C. (2007). Magnetic properties of a noninterpenetrating chiral porous cobalt metal-organic framewok. J. Appl. Phys..

[64] Lee Y.M., Song Y.J., Poong J.I., Kim S.H., Koo H.G., Lee J.A., Kim C., Kim S.-J., Kim Y. (2010). Novel chains constructed from heterotrinuclear units and 1,2-bis(4-pyridyl)ethane formulated as [M_2_M′(O_2_CPh)_6_](bpa) (M = Co, Zn, M′ = Co, Cd): Their catalytic activity. Inorg. Chem. Commun..

[65] Fan J., Gan L., Kawaguchi H., Sun W.Y., Yu K.B., Tang W.X. (2003). Reversible anion exchanges between the layered organic-inorganic hybridized architectures: Syntheses and structures of manganese(II) and copper(II) complexes containing novel tripodal ligands. Chem. Eur. J..

[66] Mu B., Huang Y., Walton K.S. (2010). A metal-organic framework with coordinatively unsaturated metal centers and microporous structure. CrystEngComm.

[67] Chin J.M., Chen E.Y., Menon A.G., Tan H.Y., Hor A.T.S., Schreyer M.K., Xu J. (2013). Tuning the aspect ratio of NH_2_-MIL-53(Al) microneedles and nanorods via coordination modulation. CrystEngComm.

[68] Hafizovic J., Bjorgen M., Olsbye U., Dietzel P.D.C., Bordiga S., Prestipino C., Lamberti C., Lillerud K.P. (2007). The inconsistency in adsorption properties and powder XRD data of MOF-5 is rationalized by framework interpenetration and the presence of organic and inorganic species in the nanocavities. J. Am. Chem. Soc..

[69] Stamatatos T.C., Boudalis A.K., Pringouri K.V., Raptopoulou C.P., Terzis A., Wolowska J., McInnes E.J.L., Perlepes S.P. (2007). Mixed-valence cobalt(II/III) carboxylate clusters: Co_4_^II^Co_2_^III^ and Co^II^Co_2_^III^ complexes from the use of 2-(hydroxymethyl)pyridine. Eur. J. Inorg. Chem..

[70] Chen W.-X., Zhuang G.-L., Zhao H.-X., Long L.-S., Huang R.-B., Zheng L.-S. (2011). Magnetic and thermal properties of three ionothermally synthesized metal-carboxylate frameworks of [M_3_(ip)_4_][EMIm]_2_ (M = Co, Ni, Mn, H_2_ip = isophthalic acid, EMIm = 1-ethyl-3-methyl imidazolium). Dalton Trans..

[71] Zhao J., Dong W.-W., Wu Y.-P., Wang Y.-N., Wang C., Li D.-S., Zhang Q.-C. (2015). Two (3,6)-connected porous metal-organic frameworks based on linear trinuclear [Co_3_(COO)_6_] and paddlewheel dinuclear [Cu_2_(COO)_4_] SBUs: Gas adsorption, photocatalytic behaviour, and magnetic properties. J. Mater. Chem. A.

[72] Dolomanov O.V., Blake A.J., Champness N.R., Schröder M. (2003). OLEX: New software for visualization and analysis of extended crystal structures. J. Appl. Cryst..

[73] Wollmann P., Leistner M., Stoeck U., Gruenker R., Gedrich K., Klein N., Throl O., Graehlert W., Senkovska I., Dreisbach F. (2011). High-throughput screening: Speeding up porous materials discovery. Chem. Commun..

[74] Spek A.L. (2003). Single-crystal structure validation with the program PLATON. J. Appl. Cryst..

[75] Sumida K., Rogow D.L., Mason J.A., McDonald T.M., Bloch E.D., Herm Z.R., Bae T.-H., Long J.R. (2012). Carbon dioxide capture in metal-organic frameworks. Chem. Rev..

[76] Liu J., Thallapally P.K., McGrail B.P., Brown D.R., Liu J. (2012). Progress in adsorption-based CO_2_ capture by metal-organic frameworks. Chem. Soc. Rev..

[77] Li J.-R., Kuppler R.J., Zhou H.-C. (2009). Selective gas adsorption and separation in metal-organic frameworks. Chem. Soc. Rev..

[78] Feldblyum J.I., Liu M., Gidley D.W., Matzger A.J. (2011). Reconciling the discrepancies between crystallographic porosity and guest access as exemplified by Zn-HKUST-1. J. Am. Chem. Soc..

[79] Liu M., Wong-Foy A.G., Vallery R.S., William E., Frieze J., Schnobrich K., Gidley D.W., Matzger A.J. (2010). Evolution of nanoscale pore structure in coordination polymers during thermal and chemical exposure revealed by positron annihilation. Adv. Mater..

[80] Li J.-R., Ma Y., McCarthy M.C., Sculley J., Yu J., Jeong H.-K., Balbuena P.B., Zhou H.-C. (2011). Carbon dioxide capture-related gas adsorption and separation in metal-organic frameworks. Coord. Chem. Rev..

[81] Armaghan M., Shang X.J., Yuan Y.Q., Young D.J., Zhang W.H., Hor T.S.A., Lang J.P. (2015). Metal-organic frameworks via emissive metal-carboxylate zwitterion intermediates. ChemPlusChem.

[82] Chen J.-X., Zhao H.-Q., Li H.-H., Huang S.-L., Ding N.-N., Chen W.-H., Young D.J., Zhang W.-H., Andy Hor T.S. (2014). Bent tritopic carboxylates for coordination networks: Clues to the origin of self-penetration. CrystEngComm.

[83] Chen J.-X., Chen M., Ding N.-N., Chen W.-H., Zhang W.-H., Hor T.S.A., Young D.J. (2014). Transmetalation of a dodecahedral Na_9_ aggregate-based polymer: A facile route to water stable Cu(II) coordination networks. Inorg. Chem..

[84] Sing K.S.W., Williams R.T. (2004). The use of molecular probes for the characterization of nanoporous adsorbents. Part. Part. Syst. Charact..

[85] Moellmer J., Celer E.B., Luebke R., Cairns A.J., Staudt R., Eddaoudi M., Thommes M. (2010). Insights on adsorption characterization of metal-organic frameworks: A benchmark study on the novel soc-MOF. Microporous Mesoporous Mater..

[86] Moon H.R., Kobayashi N., Suh M.P. (2006). Porous metal-organic framework with coordinatively unsaturated Mn^II^ sites: Sorption properties for various gases. Inorg. Chem..

[87] Sheldrick G.M. (1996). SADABS: Program for Empirical Absorption Correction of Area Detector Data.

[88] Sheldrick G. (2015). Crystal structure refinement with *SHELXL*. Acta Crystallogr. Sect. C.

[89] Spek A.L. (2015). *PLATON* SQUEEZE: A tool for the calculation of the disordered solvent contribution to the calculated structure factors. Acta Crystallogr. Sect. C.

[90] Zhu L., Sheng D., Xu C., Dai X., Silver M.A., Li J., Li P., Wang Y., Wang Y., Chen L. (2017). Identifying the recognition site for selective trapping of ^99^TcO_4_^−^ in a hydrolytically stable and radiation resistant cationic metal-organic framework. J. Am. Chem. Soc..

[91] Desai A.V., Manna B., Karmakar A., Sahu A., Ghosh S.K. (2016). A water-stable cationic metal-organic framework as a dual adsorbent of oxoanion pollutants. Angew. Chem. Int. Ed..

