# Supplementary material for: Co2 and Co3 Mixed Cluster Secondary Building Unit Approach toward a Three-Dimensional Metal-Organic Framework with Permanent Porosity
Source: Molecules. 2018 Mar 25;23(4):755. doi: 10.3390/molecules23040755 (PMC6017799; doi:10.3390/molecules23040755)
Supplement: Supplementary file 1 [file molecules-23-00755-s001.pdf]

Co_2 and Co_3 Mixed Cluster Secondary Building Unit Approach toward a Three-Dimensional Metal-Organic Framework with Permanent Porosity

Meng-Yao Chao, Wen-Hua Zhang * and Jian-Ping Lang *

College of Chemistry, Chemical Engineering and Materials Science, Soochow University, Suzhou 215123, China; 15062328714@163.com

* Correspondence: whzhang@suda.edu.cn (W.-H.Z.); jplang@suda.edu.cn (J.-P.L.); Tel.: Tel.: 86-512-65883615 (W.-H.Z.)

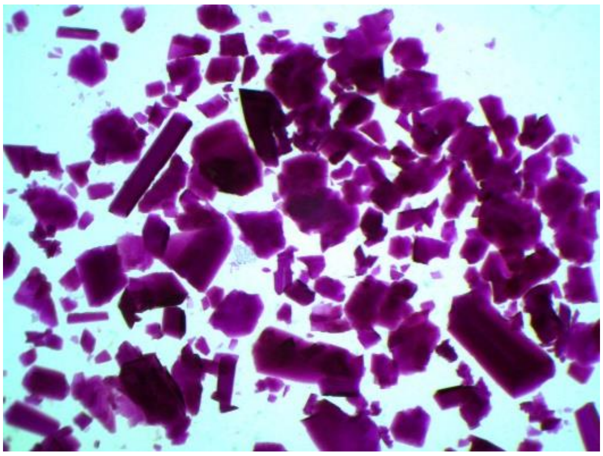

Figure S1. The photo of as-synthesized MOF **1** crystals.

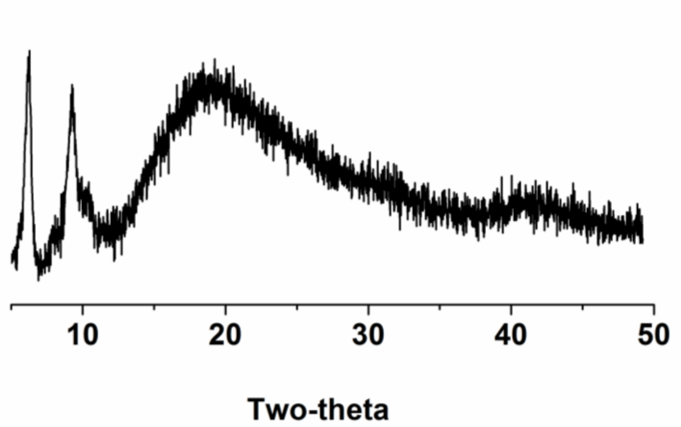

Figure S2. The PXRD pattern of MOF **1** after BET test showing its amorphous nature.

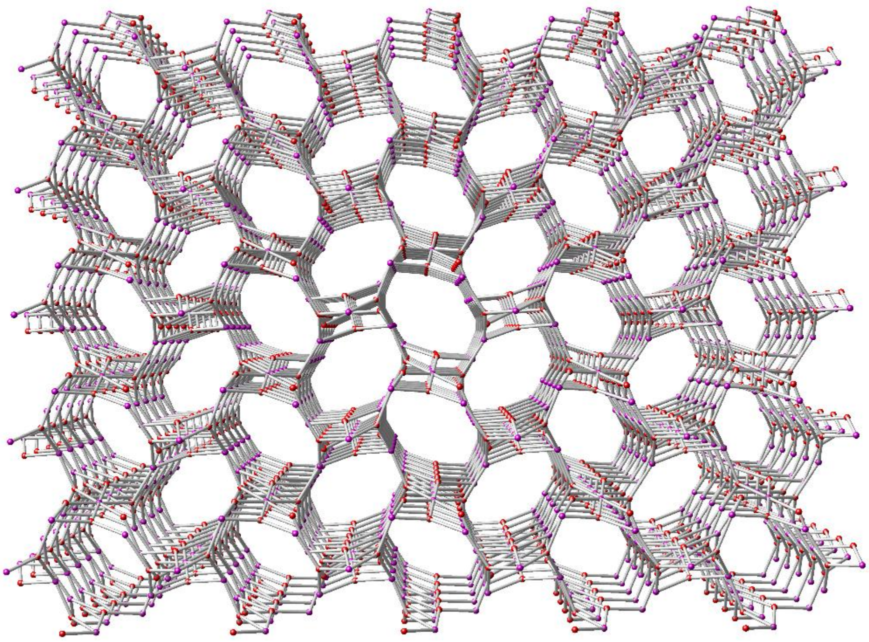

Figure S3. The topological network of MOF **1** by considering the Co_3 SBU as a 6-connecting node, while the two types of Co_2 SBUs, as well as three independent BTB ligands as 3-connecting nodes.
